# Cross-domain cooperation drives nutrient acquisition and metabolism in the bark beetle holobiont

**DOI:** 10.1093/ismejo/wrag227

**Published:** 2026-09-16

**Authors:** Zaki Saati-Santamaría, Tereza Veselská, Karel Švec, Martin Kostovčík, Ezequiel Peral-Aranega, Barbora Křížková, Paula García-Fraile, Miroslav Kolařík

**Affiliations:** Laboratory of Fungal Genetics and Metabolism, Institute of Microbiology, The Czech Academy of Sciences, Prague 142 00, Czech Republic; Institute for Agribiotechnology Research (CIALE), Universidad de Salamanca, Salamanca 37004, Spain; Departamento de Microbiología y Genética, Universidad de Salamanca, Salamanca 37004, Spain; Unidad de Excelencia Producción, Agrícola y Medioambiente (AGRIENVIRONMENT), Universidad de Salamanca, Salamanca 37004, Spain; Laboratory of Fungal Genetics and Metabolism, Institute of Microbiology, The Czech Academy of Sciences, Prague 142 00, Czech Republic; Laboratory of Fungal Genetics and Metabolism, Institute of Microbiology, The Czech Academy of Sciences, Prague 142 00, Czech Republic; Laboratory of Fungal Genetics and Metabolism, Institute of Microbiology, The Czech Academy of Sciences, Prague 142 00, Czech Republic; Institute for Agribiotechnology Research (CIALE), Universidad de Salamanca, Salamanca 37004, Spain; Departamento de Microbiología y Genética, Universidad de Salamanca, Salamanca 37004, Spain; Laboratory of Fungal Genetics and Metabolism, Institute of Microbiology, The Czech Academy of Sciences, Prague 142 00, Czech Republic; Institute for Agribiotechnology Research (CIALE), Universidad de Salamanca, Salamanca 37004, Spain; Departamento de Microbiología y Genética, Universidad de Salamanca, Salamanca 37004, Spain; Unidad de Excelencia Producción, Agrícola y Medioambiente (AGRIENVIRONMENT), Universidad de Salamanca, Salamanca 37004, Spain; Associated Research Unit of Plant–Microorganism Interaction, Universidad de Salamanca—IRNASA–CSIC, Salamanca 37004, Spain; Laboratory of Fungal Genetics and Metabolism, Institute of Microbiology, The Czech Academy of Sciences, Prague 142 00, Czech Republic

**Keywords:** holobiont, host–microbe interactions, co-metabolism, symbiosis, metatranscriptome, cross-domain RNA-Seq, bark beetle, metabolic complementarity, microbial cooperation

## Abstract

Microbial symbiosis underpins host adaptation, yet mechanisms of metabolic integration in holobionts remain unclear. Using metatranscriptomics, genomics, and metabolic assays, we investigated gut microbiome interactions in the European spruce bark beetle (*Ips typographus*). We observed metabolic complementarity among symbionts and host, forming cross-domain networks that support nutrient acquisition. Nitrogen recycling revealed strong interdependence: no single partner possessed a complete uric acid degradation pathway, but combined evidence supports a distributed pathway spanning beetle, bacteria, and fungi. Additionally, bacterial nitrate reduction to ammonia indicates a potential nitrogen influx, making otherwise inaccessible inorganic nitrogen available to the host. Shaped by microbial interactions, symbionts also likely supply specific amino acids, while vitamin metabolism showed cross-domain co-metabolism, with bacteria as main producers of B vitamins, while host and fungi modulated interconversion. Carbohydrate degradation was highly partitioned; bacteria target xylan and pectin, while fungi contribute to glucan breakdown. Crucially, our data provide indirect evidence that the beetle may contribute to complete cellulose degradation, highlighting an underappreciated host role in lignocellulose processing. In terms of enzymatic functional diversity, the bacteriome emerged as the most important microbiome component—an observation that contrasts with the traditional focus on fungi and underscores the need to consider bacterial contributions in insect symbioses. Despite life-stage variation, core metabolic functions remained stable. Overall, metabolic interdependence, rather than microbial composition alone, structures holobiont function. These results highlight functional redundancy and ecological resilience, emphasizing the importance of microbial cooperation and host–microbe metabolic evolution.

## Introduction

The intricate relationships between microorganisms and their hosts highlight the significance of metabolic integration within their shared ecosystems [1]. This integration contributes to several vital ecological processes, including reproduction, defense against pathogens, tolerance to environmental stress, and the degradation of complex nutrients (e.g. plant polymers), enabling the use of diverse resources and promoting mutualistic interactions [2–5]. Such relationships are shaped by environmental filtering, where abiotic and biotic factors select for specific traits or genes that enable survival and adaptability in a given context, reflecting the dynamics of evolutionary processes [6, 7]. This highlights the role of microbial symbionts as essential contributors to the ecological functions of holobionts, as their combined metabolic activities not only support nutrient cycling but also enhance the host’s ability to exploit its environment [1, 8].

The metabolic pathways shared among organisms cohabiting in a niche, including hosts and microorganisms, exemplify how symbiotic associations can evolve to optimize resource utilization [9–11]. The interdependence observed in these relationships may further shape the evolutionary trajectories of the species involved, consistent with the *Black Queen Hypothesis* [12], with symbiotic partnerships serving as a critical mechanism for resilience in fluctuating environments [13]. This collaboration between microbial communities and their host not only influences the functional dynamics of their ecosystems but also provides insights into the evolutionary pressures that drive the diversification and specialization of metabolic functions in response to environmental challenges [14–16].

Here, we use the European spruce bark beetle (ESBB) (*Ips typographus*—Coleoptera, Curculionidae, Scolytinae) as a model organism to examine how microbial symbionts contribute to nutrient acquisition and environmental adaptation. *Ips typographus* is not only a keystone species in Eurasian spruce forests but also the most economically important forest pest in the Palearctic region [17]. During outbreak years, its populations can reach densities capable of overwhelming even healthy spruce trees, resulting in extensive tree mortality. Recent climate-driven events, such as droughts and storms, have exacerbated this impact—recent outbreaks have caused the loss of >40 million m^3^ of wood in Europe [17, 18]. The life cycle of *I. typographus* involves adult males boring into the inner bark to establish mating chambers, after which females construct oviposition galleries where they deposit eggs; the developing larvae feed within the phloem, a nutritionally imbalanced environment where microbial symbionts may play a crucial role [18, 19]. Although *I. typographus* is a significant pest in European coniferous forests [17], it may also serve as a powerful model to investigate microbial cooperation and functional diversity within a holobiont. Bark beetles rely on their symbionts to overcome the nutritional challenges posed by their diet of bark and phloem. Although this tissue can contain substantial carbohydrate reserves—such as sucrose and starch stored in parenchyma cells—these compounds may require enzymatic processing to become accessible, a task in which microbial partners likely contribute. Moreover, phloem is relatively poor in freely available nitrogen sources, essential amino acids, and certain vitamins [20–23]. Many insects lack the metabolic capacity to synthesize essential amino acids *de novo*, as well as several B vitamins, and endogenous metabolism alone may not be sufficient to meet the demand for certain nutrients under nutrient-limited conditions [24–26]. As in other insects, in the ESBB system these key biomolecules can be obtained from plant tissues, but could also be synthesized or salvaged by microbial symbionts, thereby playing a central role in supporting the beetle’s survival and development [24, 25, 27].

Research on the microbiome of *I. typographus* and other European spruce bark beetles (ESBB) has primarily focused on community composition. This beetle harbors a core microbiome comprising filamentous fungi, yeasts, and bacteria [28], including several *Ophiostoma* and *Grosmannia* species, *Ceratocystiopsis minuta, Ceratocystis picea, Endoconidiophora polonica, Leptographium piceaperdum*, and *Rhexographium fimbriasporum*, saccharomycetous yeasts (*Wickerhamomyces, Kuraishia, Nakazawaea, Yamadazyma*, and *Cyberlindnera*), Sordariomycetes fungi (*Ophiostoma* and *Endoconidiophora*), and Gammaproteobacteria (*Erwinia, Pseudomonas, Serratia, Stenotrophomonas*, and *Pseudoxanthomonas*) [29–36]. However, the functional roles of the *I. typographus* microbiota remain underexplored. Most current insights derive from *ex vivo* assays or genome analyses of microbial isolates [37–40], which provide only a partial view of microbial activity within the host. In contrast, recent advances such as metatranscriptomics allow *in situ* exploration of host–microbe interactions and shared metabolic functions, enabling researchers to link microbial composition to real-time metabolic contributions *in vivo* [41]. Unlike static genomic or culture-based approaches, it captures active gene expression from both host and microbiota, offering a dynamic and more accurate view of functional processes shaping the holobiont. This is particularly valuable for assessing functional complementarity, as it reveals not only which genes are present, but which are actually used under specific conditions—information that cannot be inferred from genomic data alone.

We hypothesize that the *I. typographus* holobiont forms a functionally integrated system in which the beetle and its microbial partners jointly compensate for metabolic deficiencies through complementary biosynthetic capabilities. By exploring the symbiotic relationships revealed through its gut metatranscriptome, we aim to uncover the mechanisms of metabolic cooperation within this system. Our findings seek to enhance understanding of how microbial communities contribute to their host’s survival strategies in nutrient-poor environments, highlighting their role in biosynthetic pathways essential for adaptation and resilience. This knowledge provides insights into how such partnerships drive specialization and ecological success in challenging ecosystems.

## Materials and methods

### Sample collection

Samples analyzed in this study were collected simultaneously and from the same locations as those used in our previous work [29], which characterized the core gut microbiome of *I. typographus*. In brief, four and five freshly infested spruce trees (*Picea abies*) were felled in May and August 2020, respectively, in a spruce monocultural forest in the surroundings of Nižbor city (central Czechia region, 49°59′09.9″ N 13°56′47.5″ E, 390 m.a.s.l.). Trunks were cut into logs and transported to the Institute of Microbiology of the Czech Academy of Sciences, Prague (50°1′2.262″ N 14° 27′ 52.4772″ E, 310 m.a.s.l.), where they were incubated outdoors, simulating the original forest site (same climatic zone as sampling site). This setup was selected to ensure consistent access to material for developmental staging and sampling, while preserving environmental conditions similar to the natural forest. While some microenvironmental changes may occur, we consider this a reliable semi-natural approximation to field conditions. Under these conditions, multiple overlapping colonization and oviposition events can occur within and among logs, resulting in galleries that may differ slightly in their initiation time even within the same incubation period.

To capture the broadest possible range of gene activity and reconstruct the metabolism of the holobiont more comprehensively, we sampled insects at different life stages. Parental adults were collected in the first 2 days, first instar larvae on the 5th–10th day, and teneral adults on the 15th–20th day a of log incubations. All samples consisted of pooled individuals from multiple galleries and were not derived from a single tracked cohort, but rather reflect the natural heterogeneity of gallery development under field-like conditions. We sampled insect individuals regardless of their sex and beetle species determination was based on their external morphological characteristics under a binocular magnifier (Nikon SZ30, Minato, Japan) using taxonomic literature [42]. Samples were surface disinfected after the removal from the gallery system by three subsequent washes with 70% ethanol, 2% Tween 80 (Avantor, USA), and sterile distilled water. To increase microbial to beetle biomass proportion, we eviscerated the whole gut of each specimen under the binocular magnifier directly into lysis buffer RA1 (Nucleospin RNA plant kit; Macherey-Nagel) supplemented with 1% β-mercaptoethanol. One sample consisted of 10 guts. In total, we obtained seven samples from parental adults, six samples of larvae L1, and five samples of teneral adults. Pupae and eggs were not analyzed as the softness of its surfaces impeded such manipulation. Pooled samples were frozen at −80°C in Eppendorf tubes till RNA extraction, if necessary.

### RNA extraction

RNA extraction was performed using the Nucleospin RNA Plant Kit (Macherey-Nagel). Samples were lysed in RA1 buffer, supplemented with 1% β-mercaptoethanol, and stored at −80°C until further processing. Homogenization was achieved using Lysing Matrix A beads (MP Biomedicals) in a FastPrep-24™ 5G Instrument (Irvine, CA, USA) at a speed of 5.5 m/s for 30 s. The DNA digestion step in the kit’s protocol was skipped initially and later carried out using the TURBO DNA-free kit (Invitrogen). PCR amplification of ITS and 16S rRNA gene regions followed by agarose gel electrophoresis confirmed the absence of residual DNA. RNA purity was assessed using a NanoDrop™ 2000c spectrophotometer (Thermo Fisher Scientific), and samples with *A*_260_/*A*_280_ and *A*_260_/*A*_230_ ratios <1.8 underwent additional purification through isopropanol precipitation with 3 M sodium acetate (pH 5.2). RNA concentrations were determined with the Qubit RNA BR Assay Kit on a Qubit 2.0 Fluorometer (Thermo Fisher Scientific). RNA quality was further evaluated using a Bioanalyzer with the RNA 6000 Pico Kit (Agilent). RNA concentrations were measured with the Qubit RNA High Sensitivity Assay Kit (Thermo Fisher Scientific).

### Library construction and sequencing

cDNA libraries were prepared using the Zymo-Seq RiboFree Total RNA-Seq Library Kit (Zymo Research), adhering to the manufacturer’s protocol. The quality of the libraries was verified using the High Sensitivity DNA Kit on a Bioanalyzer (Agilent). Sequencing was performed at the CEITEC Institute (Brno, Czechia) on the NovaSeq 6000 System (Illumina), generating 2 × 150 base pair (bp) paired-end reads.

### Metatranscriptome analysis

Raw data were processed using SqueezeMeta (v1.6.2) [43]. Within this pipeline, the assembly was done using SPAdes (v3.15.5) [44] whereby short contigs (<200 bp) were removed and contig statistics produced using prinseq [45]. Open reading frames (ORFs) were predicted using Prodigal [46]. Similarity searches for GenBank nr [47], which was used to get the taxonomic annotations, and for eggnog(v4) [48], and KEGG [49] were done using Diamond (v2.0.15) [50]. HMM homology searches were done by HMMER (v3.1b2) [51] for the Pfam database [52]. Additional ORFs were obtained by Diamond BlastX [50]. Read mapping against contigs was performed using Bowtie2 (v2.4.1) [53]. The abundance of the genes was normalized based on contig/gene length and sampling depth, to finally get transcripts per million (TPM). Pathway prediction for KEGG [49] and MetaCyc [54] databases was done using MinPath (v1.21) [55].

Differential gene expression analysis was conducted in DESeq2 (v1.44.0) [56] in R (v4.4.0). The dataset used as input excluded non-microbial (Viridiplantae and Metazoa) sequences. Gene-level counts were normalized using the DESeq2 default method, which estimates size factors to account for sequencing depth differences across samples. Differential expression analyses were conducted across three biological conditions—larva, teneral adult, and parental adult—by testing three pairwise contrasts. For each contrast, log_2_ fold change values and Benjamini–Hochberg adjusted *P* values (padj) were extracted.

To explore global expression shifts across life stages, ternary plots were generated using the ggtern package (v3.5.0) [57]. For each gene, expression proportions in the three conditions were calculated based on normalized means. Genes were assigned to differential abundance categories (DAA) if they exhibited a padj <.05 in at least one contrast. Each gene was assigned to a single DAA group corresponding to the first contrast that met the significance threshold, enabling visualization of predominant expression shifts between conditions. The ternary plot displayed gene expression bias across stages, with point color reflecting DAA category, point size representing log-transformed mean expression, and alpha transparency indicating statistical non-significance.

### KEGG enrichment analysis

To assign functional roles to differentially abundant genes, KEGG Orthology (KO) annotations were mapped to ORFs using a manually curated annotation table derived from prior metatranscriptomic analyses. The annotation dataset excluded non-microbial (Viridiplantae and Metazoa) sequences and retained only ORFs with valid KEGG IDs. ORF-to-KO mappings were used to generate a custom TERM2GENE table for enrichment analysis.

Functional enrichment of KEGG terms was performed using the enricher() function from the clusterProfiler R package (v4.12.6) [58]. Differentially abundant genes from the larva versus parental adult comparison were selected based on an adjusted *P*-value <.05 and an absolute log_2_ fold change >1. These genes were used as input for a hypergeometric test against the full set of annotated ORFs. KEGG categories with enrichment *P*-values <.05 were retained for interpretation. Only enrichment results for the larva versus parental adult contrast are presented in the manuscript, as this comparison captured the most pronounced transcriptomic differences across life stages.

### Endoglucanases phylogeny

To investigate the phylogenetic relationships of glycoside hydrolases (GHs) with putative endo-β-1,4-glucanase activity (EC 3.2.1.4), sequences were extracted from the HMMER annotations generated by run_dbcan3 [59]. The target families included GH5, GH6, GH7, GH8, GH9, GH10, GH12, GH26, GH44, GH45, GH51, GH74, GH124, and GH148, as defined by the CAZy database (https://www.cazy.org/; accessed on 23 October 2024) [60]. Extracted sequences were collated into a single FASTA file for subsequent analyses. Sequences were aligned using MAFFT (v7) [61] with automatic alignment settings. The resulting multiple sequence alignment was processed with TrimAl (v1.4.rev15) [62] to remove poorly aligned regions and produce a refined alignment using the -automated1 trimming mode. Phylogenetic reconstruction was conducted using IQ-TREE (v2.2.0) [63] with ModelFinder Plus (MFP) to determine the optimal substitution model and ultrafast bootstrap (UFBoot) with 1000 replicates for branch support. Branch support was further evaluated using SH-like approximate likelihood-ratio tests with 1000 replicates. Because these GHs might have enzymatic activities beyond that of endo-β-1,4-glucanase, we searched for the functional annotation of these GH proteins in the SqueezeMeta output, and we labeled in the phylogenetic tree those annotated as endoglucanases.

### Riboflavin quantification by UHPLC–MS

Riboflavin (vitamin B_2_) production by bacterial isolates from *I. typographus* was evaluated under vitamin-depleted conditions. A bacterial preculture was prepared in 4 ml of modified M9 medium lacking B vitamins, but supplemented with trace elements (1 ml/L), mannitol (10 g/L), and 0.1% (w/v) casamino acids (added after autoclaving by sterile filtration), in airtight 10-ml tubes. After adjusting the optical density at 600 nm (OD600) to 0.1 with sterile PBS, cultures were incubated at 28°C and 180 rpm in the dark until reaching an OD600 >1 (24–48 h depending on the strain). Cells were pelleted by centrifugation (6000 rpm, 10 min, 4°C), washed twice with PBS, and resuspended in fresh modified M9 to an OD600 of 0.1. Final cultures were incubated for 5 days under the same conditions. Supernatants were harvested by centrifugation, filtered (0.22 μm), and stored at −20°C until analysis.

Riboflavin was quantified at the Elemental Analysis, Chromatography and Mass Spectrometry Service (NÚCLEUS), University of Salamanca (Spain). The analysis was carried out using a Vanquish UHPLC system coupled to a UV detector and an Orbitrap Q-Exactive Focus mass spectrometer (Thermo Scientific™, USA), equipped with a commercial Ascentis® Express C18 column (Sigma-Aldrich, Germany).

Quantification was performed using full-scan MS acquisition mode (Full MS), with the following mass spectrometry parameters: *m/z* range from 100 to 500, mass resolution of 70 000 (at *m/z* 200), AGC target of 1 × 10^6^, and one microscan per spectrum.

The chromatographic separation was achieved using a gradient of methanol and water containing 0.1% formic acid. The gradient was set as follows: 5% methanol for the first 10 min, increased to 40% by minute 15, and ramped to 100% by minute 20, held constant until minute 25. The gradient then returned to 5% at minute 26 and was maintained until the end of the run (minute 30). The flow rate was 200 μl/min, and the injection volume was 10 μl.

A riboflavin standard solution at 1 ppm (purity >98.0%; Glentham Life Sciences Ltd., UK) was used for retention time calibration. The detection limit (LOD) for riboflavin was 5 ppb.

### Growth on different nitrogen sources

The ability of bacterial and fungal isolates to utilize alternative nitrogen sources was assessed using solid minimal media supplemented with different nitrogen compounds. Media were solidified with agarose (1.5%–2% w/v) and prepared using either yeast carbon base (Biolife Italiana, Milan, Italy) for fungal isolates or vitamin-free JMM medium [64] for bacterial isolates. Nitrogen sources included allantoin and uric acid, while ammonium nitrate was additionally used as a positive control for fungal assays. Nitrogen compounds were dissolved in distilled water, adjusted to pH 7 when necessary, sterilized by filtration (0.22 μm), and added to autoclaved medium cooled to ~50°C. Media lacking any nitrogen source were used as negative controls.

For fungal assays, nitrogen sources were added at concentrations standardized to provide a final atomic nitrogen content of 0.108 g N per 100 ml of medium [65]. Media were dispensed into 12-well culture plates (2 ml per well). Fungal strains were pre-cultured for 2 weeks on malt extract agar or yeast peptone dextrose agar, depending on the taxonomic group. To minimize the influence of intracellular nitrogen reserves accumulated during growth on rich media, isolates were first inoculated onto the test media and incubated for 1 week at 24°C in the dark, followed by transfer to a second set of fresh plates containing the same nitrogen source.

For bacterial assays, JMM medium was supplemented with 0.1% (w/v) allantoin, uric acid, or ammonium nitrate. Isolates were streaked onto plates in triplicate and incubated at 28°C for 1 week before being transferred to fresh plates containing the same nitrogen source.

Growth was evaluated after the second transfer. Isolates showing sustained growth on media containing allantoin or uric acid were considered capable of utilizing these compounds as nitrogen sources, whereas growth on nitrogen-free medium was used to assess the ability to grow in the absence of supplemented nitrogen.

### Genome sequencing, assembly, and annotation

Genomic DNA from bacterial strains isolated from *I. typographus* [30] was extracted using the Zymo Quick-DNA Fungal/Bacterial Miniprep Kit (Zymo Research, USA), following the manufacturer’s instructions. DNA concentration and quality were assessed by fluorometric quantification using a Qubit fluorometer (Thermo Fisher Scientific, USA). Sequencing libraries were prepared using the Nextera DNA XT Library Preparation Kit (Illumina, USA) and sequenced on an Illumina NovaSeq X platform, generating 150-bp paired-end reads.

Raw sequencing reads were processed following the workflow previously described [66]. Briefly, adapter sequences, low-quality bases, and short reads (<50 nt) were removed, and only reads with a Phred quality score of at least 30 were retained for downstream analyses. The resulting high-quality reads were assembled de novo with SPAdes v4.2.0 [44]. Structural genome annotation was performed with Prokka, and functional annotation was subsequently conducted using KofamKOALA [67] to assign KEGG Orthology (KO) identifiers and metabolic functions.

We also used fungal genomes from strains isolated from *I. typographus*–*Cryptococcus porticicola* VH57_BW (GCA_024271845.1), *Kuraishia molischiana* VH39_BB (GCA_024271875.1), *Nakazawaea ambrosiae* VH60_CP (GCA_024271865.1), *Ogataea ramenticola* VH64_CZ (GCA_024271885.1), and *Wickerhamomyces bisporus* VH7_F (GCA_02-4271855.1). Gene prediction was carried out using funannotate (v1.8.17; https://funannotate.readthedocs.io/en/latest/), combining *ab initio* gene prediction tools (AUGUSTUS, SNAP, GlimmerHMM) with BUSCO-based training (*Dikarya* lineage) to accurately identify protein-coding regions in fungal genome assemblies. KofamKOALA [67] was used to retrieve KO identifiers and metabolic functions.

### Statistics and graphical representation

Heatmaps, volcano plots, and barplots were drawn with ggplot2 package (v3.5.1) [68] for R. Functional comparisons were visualized with the UpSetR package (v1.4.0) [69]. The phylogenetic tree was rerendered with iTOL (v7) [70].

TPM (transcripts per million) data belonging to microbial taxa were used to perform a non-metric multidimensional scaling (NMDS) analysis based on Bray–Curtis distances. Samples were grouped into three biological categories (larvae, parental adults, and teneral adults) according to the experimental design. NMDS dimensionality was set to two dimensions, and 100 iterations were performed to ensure convergence. The resulting NMDS coordinates were visualized with group-specific ellipses in a plot generated using ggplot2 (v3.5.1) [68]. Additionally, the variability explained by the groups was assessed using a PERMANOVA (*adonis2*, vegan v2.6-8) with 999 permutations to determine statistical significance [71, 72]. To identify specific differences between groups, pairwise PERMANOVA tests were conducted with the *adonis2* function.

## Results

### Metatranscriptome assembly and taxonomic composition

**Figure 1:**
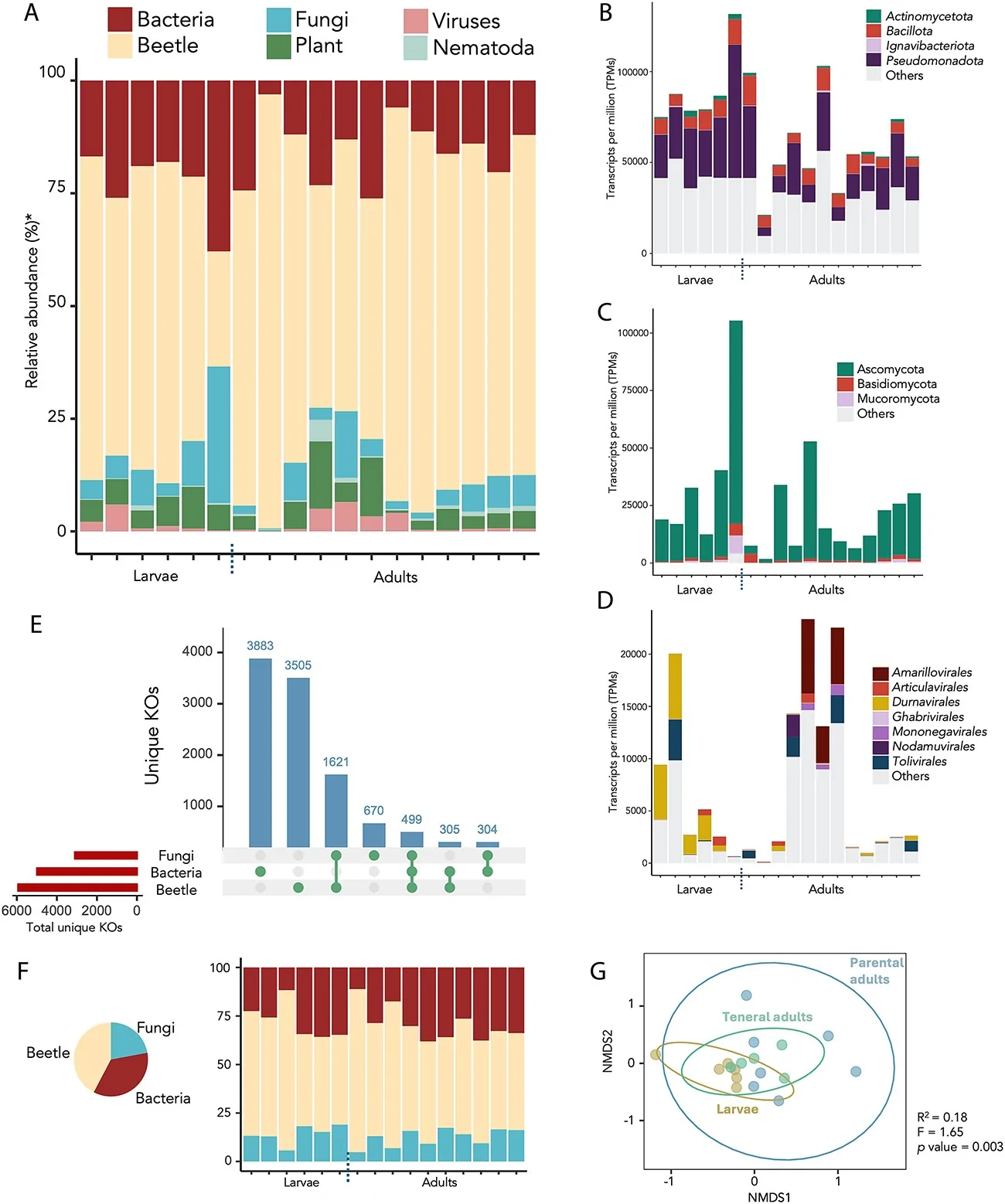
Microbial taxonomic and functional diversity in the *Ips typographus* gut metatranscriptomic dataset. Proportion of metatranscriptomic reads belonging to different taxonomic groups (a), or the main bacterial (b), fungal (c), and viral (d) phyla per sample. (e) The total count of non-redundant KOs active in the data is represented by the horizontal bars, categorized into three groups: beetle, bacteria, and fungi. The vertical bars display the distribution of shared non-redundant functions between these groups (indicated by connected green dots) or functions unique to a specific taxonomic group (indicated by unconnected dots). (f) Total count of non-redundant KOs in the data per sample, categorized into the same three taxonomic groups. (g) Non-metric multidimensional scaling (NMDS) ordination of the samples by the beetle life stage, representing only fungal and bacterial contributions. Statistic values were calculated with PERMANOVA (permutations = 999).

After quality filtering, a total of 848 680 032 reads were processed, resulting in the assembly of 611 649 contigs with a combined length of 416 992 264 base pairs. A total of 602 218 ORFs were identified, of which 175 002 received KEGG annotations, 254 522 were classified within COG categories, and 111 918 were linked to Pfam domains. Our metatranscriptomic analysis captured a significant representation of transcripts from bacteria (16.8%), fungi (6.1%), and viruses (1.7%) (Fig. 1A), providing key insights into the microbial components of the bark beetle holobiont. Moreover, 70.6% of the reads were mapped to *Arthropoda*, reflecting the beetle’s gut transcriptome. Additionally, 4.8% of the transcripts were derived from *Viridiplantae*, likely originating from ingested plant material, though these were not further analyzed in this study. Also, *Nematoda* transcripts were consistently found in the dataset, with a low representation (0.7%). Most bacterial activity was attributed to *Pseudomonadota* and *Bacillota*, with a lower proportion of transcripts assigned to *Actinomycetota* and *Ignavibacteriota* (Fig. 1B). Fungal transcripts were predominantly from Ascomycota, with Basidiomycota detected at very low levels and Mucoromycota nearly absent, along with other less abundant phyla (Fig. 1C). Therefore, in this manuscript, we will primarily focus on the active functions of Ascomycota to minimize biases associated with incomplete pathways from less abundant taxa. Viral transcripts exhibited a high diversity of phyla, including mycoviruses and bacteriophages, with no consistent taxonomic patterns (Fig. 1D).

The beetle transcriptome represents the most abundant component in our dataset, while bacterial and fungal transcripts contribute a comparable level of functional diversity (Fig. 1E, F). This observation underscores the functional complementarity of symbionts within the holobiont. In subsequent sections, we focus on the shared metabolic contributions of microbial and host partners, highlighting core functions that are consistently expressed across life stages and support the holobiont as a whole. Although the PERMANOVA identified significant differences in microbial activity among life stages (*R*^2^ = 0.18, *P* value = .003), the relatively low *F*-statistic (*F* = 1.7) and the NMDS analysis indicate that these differences are modest compared to the variability within groups (Fig. 1G). Specifically, the “larvae” group showed modest but significant differences when compared to both parental adults (*R*^2^ = 0.15, *P* value = .008, *F* = 2.0) and teneral adults (*R*^2^ = 0.16, *P* value = .015, *F* = 1.8). However, no significant differences were observed between the two adult stages (*P* value = .226). A more detailed exploration of stage-specific functional differences is presented in a dedicated section later in the manuscript.

### Degradation of fungal and plant cell wall polymers is likely initiated by bacteria, but subsequently completed by the entire holobiont

**Figure 2:**
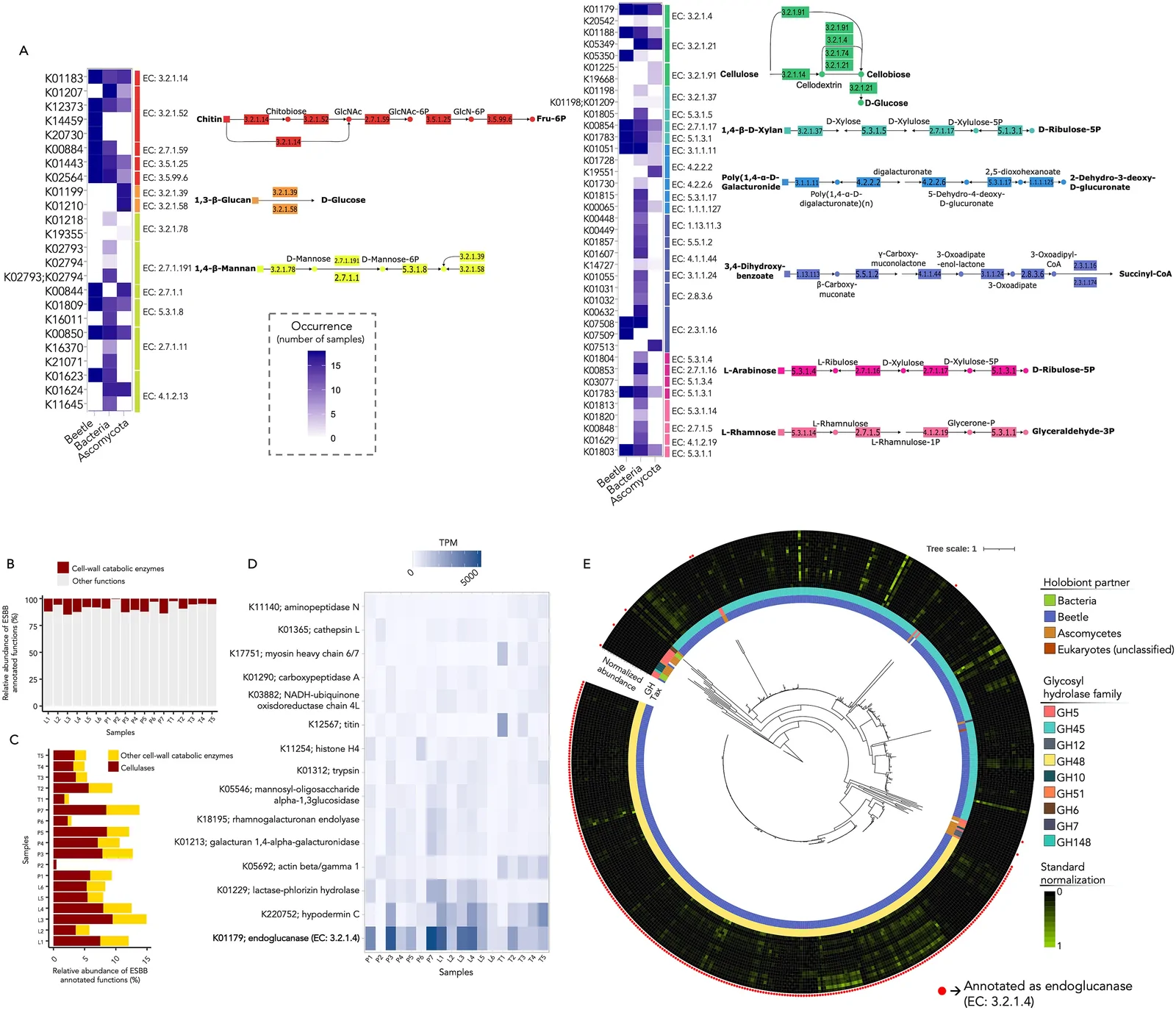
Metabolism of cell wall polysaccharides across the main groups of the beetle holobiont. (A) The heatmap shows the number of samples (*n* = 18) where each function is active (occurrence). The KO functions related to enzymatic activities (EC). These activities can be interpreted with the metabolic pathways accompanying the figure. The specific details on the occurrence of each function can be observed in Supplementary Table 1. *K00705 annotation has two potential catabolic roles: (I) starch → D-glucose 1-phosphate and (II) maltose → D-glucose + amylose. (B) Proportion of *I. typographus* transcripts involved in cell wall polysaccharide catabolic enzymes relative to their total annotated functions. (C) Proportion of *I. typographus* transcripts involved in cellulase or different cell wall polysaccharide catabolic enzymes relative to their total annotated functions. (D) Heatmap of top 15 functions with KEGG annotations counted in TPM (transcripts per million) as recovered from the full dataset. Endoglucanases (EC: 3.2.1.4) represent the most expressed annotated function in the *Ips typographus* gut. (E) Phylogeny of glycosyl hydrolases (GHs) with potential endoglucanase activity identified in the *I. typographus* gut. Proteins specifically annotated as “endoglucanases” are marked with circles. The innermost circle categorizes the proteins by their source organism (beetle, bacteria, or fungi). The middle circle represents the classification of proteins into GH families. The outer heatmap illustrates the abundance of each function (standard normalized) across the analyzed samples.

Microbial symbionts are known to participate in the degradation of diverse plant- and fungal-derived polymers which may contribute to nutrient processing within the host. However, most of these activities remain unverified *in vivo* within the beetle gut. To investigate these processes, we examined microbial transcripts associated with these pathways, exploring their potential integration with beetle metabolism (Fig. 2A, Supplementary Table 1). Due to the lack of a temporal gradient in transcriptomic data—other than differences based on insect life stages—we focused on the occurrence of functional genes across samples (i.e. the number of samples where a gene was detected and annotated), rather than on the overall abundance of these transcripts.

Our data indicate that microbial symbionts play a crucial role in plant cell wall degradation (Fig. 2A). Notably, a substantial fraction of the beetle’s own transcriptome is also devoted to this process, with cell wall–degrading enzymes accounting for an average of 8.3% of all annotated beetle transcripts (Fig. 2B). In contrast, even though both bacteria and Ascomycota contribute essential functions to this metabolism, <1% of their transcription is allocated to this process. Chitin degradation to *N*-acetylglucosamine (GlcNAc) can be carried out by all three entities of the holobiont (Ascomycota, bacteria, and beetle). The conversion of GlcNAc to GlcNAc-6P via *N*-acetylglucosamine kinase [EC:2.7.1.59] is mediated either by the beetle or bacteria, although its further conversion to GlcN-6P can be accomplished by all three entities. The final conversion to fructose-6-phosphate (Fru-6P) was detected across all members, though minor inconsistencies in detection between samples could be due to sequencing stochasticity (Fig. 2A, Supplementary Table 1).

Xylan degradation is likely initiated by bacteria, which could convert 1,4 β-d-xylan to d-xylose and subsequently to d-xylulose. This process may be further supported by the beetle and fungi. In the case of pectin, we focused on the degradation of poly(1,4-α-d-galacturonide), a key step in pectin catabolism. The expression patterns suggest contributions from all three entities to the initial transformation of poly(1,4-α-d-galacturonide) to poly(1,4-α-d-galacturonate), though bacteria appear to contribute predominantly to the subsequent steps (Fig. 2A, Supplementary Table 1).

Lignin degradation, specifically the breakdown of 3,4-dihydroxy-benzoate, is driven primarily by bacteria, with the subsequent utilization of this by-product by the eukaryotic partners (Ascomycota and the beetle). Arabinose degradation is largely bacterial, with the downstream conversion of d-xylulose-5P to d-ribulose-5P supported by all three members. A similar pattern was observed for rhamnose degradation, in which the eukaryotic partners appear to utilize bacterial catabolic products (Fig. 2A, Supplementary Table 1).

The degradation of 1,3-β-glucan to d-glucose is exclusively mediated by Ascomycota. Similarly, mannan degradation is supported by all three members, although bacteria tend to use a distinct enzyme for d-mannose phosphorylation (mannose PTS permease; EC:2.7.1.191) compared to eukaryotes using hexokinase (EC:2.7.1.1). Additionally, complementary transcriptional signatures were also observed in the degradation of other storage polysaccharides such as sucrose and starch. Both bacterial and fungal communities, as well as the beetle itself, possess the enzymes required for cellulose degradation. Ascomycota also encodes cellulose 1,4-beta-cellobiosidase (EC:3.2.1.91), which may act as an alternative to endo-1,4-beta-d-glucanase (EC:3.2.1.4), potentially enhancing the degradation of cellodextrins into cellobiose (Fig. 2A, Supplementary Table 1). While cell wall–degrading functions represent a large fraction of the beetle’s activity (Fig. 2B), cellulases (EC 3.2.1.4) are indeed among the most active ESBB enzymes involved in cell wall degradation (Fig. 2C). Concretely, highest expressed function in our dataset, measured by the combined transcription levels of several genes annotated as endoglucanases (Fig. 2D), corresponds to a cellulase enzyme. This activity was initially thought to be primarily contributed by bark beetle symbionts [39, 73–79]. However, a detailed analysis of cellulase transcripts provides evidence that cellulase activity is predominantly associated with the beetle itself, mainly through glycosyl hydrolases belonging to the families 48 (GH48) and 45 (GH45) (Fig. 2E). Several copies of GH48 have been reported in the ESBB genome, being attributed to exoglucanase activity with no further functional validation [80]. Even though several GH48-containing enzymes have been shown to have catabolic activity at cellulase ends [81, 82], this domain has also been reported to be processive endoglucanase, which accomplishes a processive degradation of cellulose chains after a previous “endo” cleavage [83, 84]. Despite all the GH48 found in the beetle transcriptome being annotated as endoglucanases, we argue that this activity should be further confirmed through experimental procedures.

Overall, we identified several lines of evidence for metabolic complementarity in the degradation of plant and fungal cell wall polymers, which is primarily orchestrated by bacteria, except for glucan degradation, which is exclusively mediated by Ascomycota, and chitin and cellulose, which could be accomplished by the three partner groups—bacteria, fungi, and the beetle. The beetle and Ascomycota also partially contribute to pectin degradation. The bacterial contribution to these processes is mainly driven by *Erwinia, Delftia*, and *Phyllobacterium*, with smaller contributions from *Serratia, Sphingomonas*, and *Pantoea*, among others. In the fungal community, the main contributor is *Ogataea*, although many functions have been broadly attributed to the yeast class Saccharomycetes (mainly Saccharomycetales) and the filamentous fungi of Sordariomycetes.

### Ammonia likely plays a primary role in nitrogen utilization by the beetle holobiont, with microbial symbionts potentially expanding access to nitrate and urea

**Figure 3:**
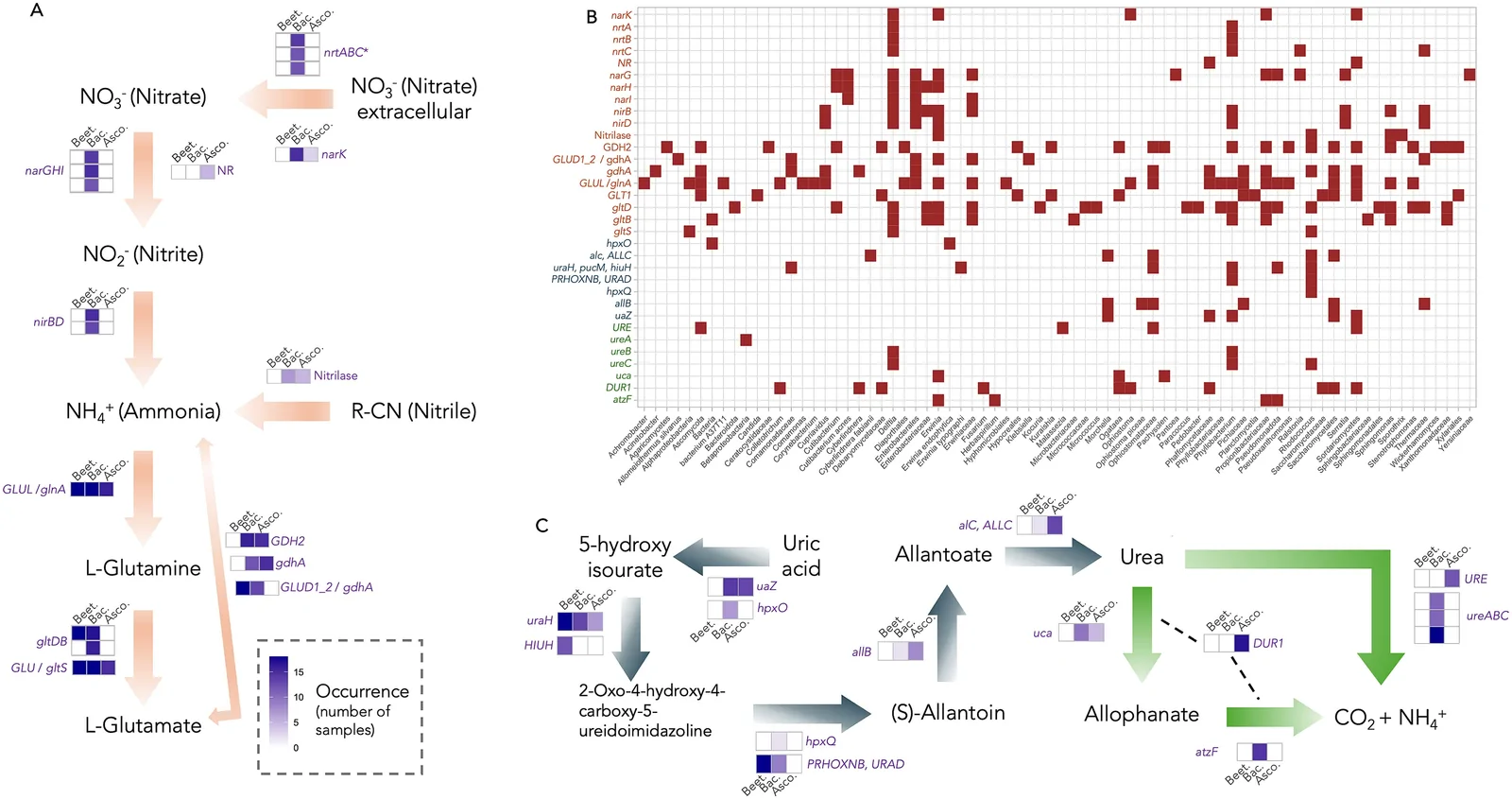
Interdependencies and co-metabolism in nitrogen metabolism within the *Ips typographus* gut. (A) Contribution of different taxonomic groups (beetle, bacteria, or fungi) to various steps in the conversion of nitrate to ammonia, followed by its incorporation into glutamine and glutamate. The heatmap indicates the number of samples (*n* = 18) in which each function is active (occurrence). The specific details on the occurrence of each function can be observed in Supplementary Table 2. (B) Diagram showing the microorganisms (classified to the most precise annotated taxonomic rank) contributing to each function represented in panels (A) and (C). (C) Contribution of different organisms (beetle, bacteria, or Ascomycota) to various steps in the conversion of urate to urea, followed by its subsequent conversion to ammonia. The heatmap shows the number of samples (*n* = 18) in which each function is active (occurrence).

Nitrogen is the primary limiting element in the ecological niche studied (*P. abies* bark). Consequently, its metabolism and acquisition by the bark beetle holobiont is a critical nutritional challenge. Here, we present evidence of diverse active pathways involved in nitrogen acquisition (Fig. 3; Supplementary Table 2). Our analysis provides evidence for nitrate uptake via nrtABC (though lacking the nrtD subunit) and its subsequent conversion to nitrite, primarily driven by bacterial taxa, including *Cutibacterium, Delftia, Erwinia*, and unclassified *Enterobacterales*, through the narGHI system. Nitrite is then reduced to ammonia exclusively by bacteria through the nirBD system, represented by *Cupriavidus, Delftia, Erwinia, Phyllobacterium, Serratia*, and unclassified *Enterobacterales* and *Thermaceae*. On the contrary, Ascomycota (*Phaffomycetaceae* and *Sordariomycetes*) express nitrate reductase in only a few samples, even though there are no signs for fungal nitrite-to-ammonia conversion. This suggests a potential scavenging role for fungi, utilizing the ammonia produced by bacterial activity (Fig. 3A, B). We did not detect any nitrogenases, enzymes that fix molecular nitrogen (N_2_).

Here, ammonia pools may also be transiently supplemented by nitrilase activity in taxa such as *Sphingomonas, Sporothrix, Rhodococcus, Erwinia*, and Ascomycota. Ammonia utilization was broadly represented across the holobiont, suggesting that it may serve as a nitrogen source for a diverse microbial taxa as well as the beetle itself. Although *Delftia* plays a major role in nitrate and nitrite reduction, transcripts encoding ammonia assimilation into l-glutamine were not detected for this taxon in our dataset, suggesting that under the conditions sampled it may rely on glutamine produced by other members of the microbiota. In contrast, taxa such as *Erwinia* expressed the complete ammonia assimilation pathway, indicating that they were actively assimilating this nitrogen source under these conditions (Fig. 3A, B).

Uric acid may also serve as an alternative nitrogen source for the holobiont, as it could be recycled either from the host’s excreted compounds or as a by-product of xanthine catabolism. Transcriptomic patterns suggest that microbial symbionts may contribute to the initial step of uric acid recycling through its conversion into 5-hydroxyurate. Subsequently, the beetle activates enzymes capable of transforming 5-hydroxyurate into allantoin, which can then be further metabolized into urea by the symbionts (Fig. 3B, C). Although we have proved that several isolates encode the complete uric acid degradation pathway (Supplementary Material), and are able to grow on uric acid or allantoin as sole nitrogen sources *in vitro* (Supplementary Table 3), no taxon—whether fungal, bacterial, or the beetle itself—activates the entire metabolic pathway in the host gut. Instead, they likely rely on one another to achieve complete uric acid recycling (Fig. 3B, C). The urea pool is later recycled by microorganisms—primarily fungi such as *Ogataea, Ophiostoma, Cyberlindnera, Kuraishia*, and *Saccharomycetaceae*, or a few bacterial taxa such as *Erwinia, Delftia*, and *Phyllobacterium*. These microorganisms actively express genes involved in the degradation of urea into ammonia and CO_2_, potentially providing more accessible nitrogen and carbon sources to the beetle holobiont. However, the beetle itself does not exhibit any gene expression related to urea utilization (Fig. 3B, C).

### Cross-domain cooperation in the amino acid metabolism

**Figure 4:**
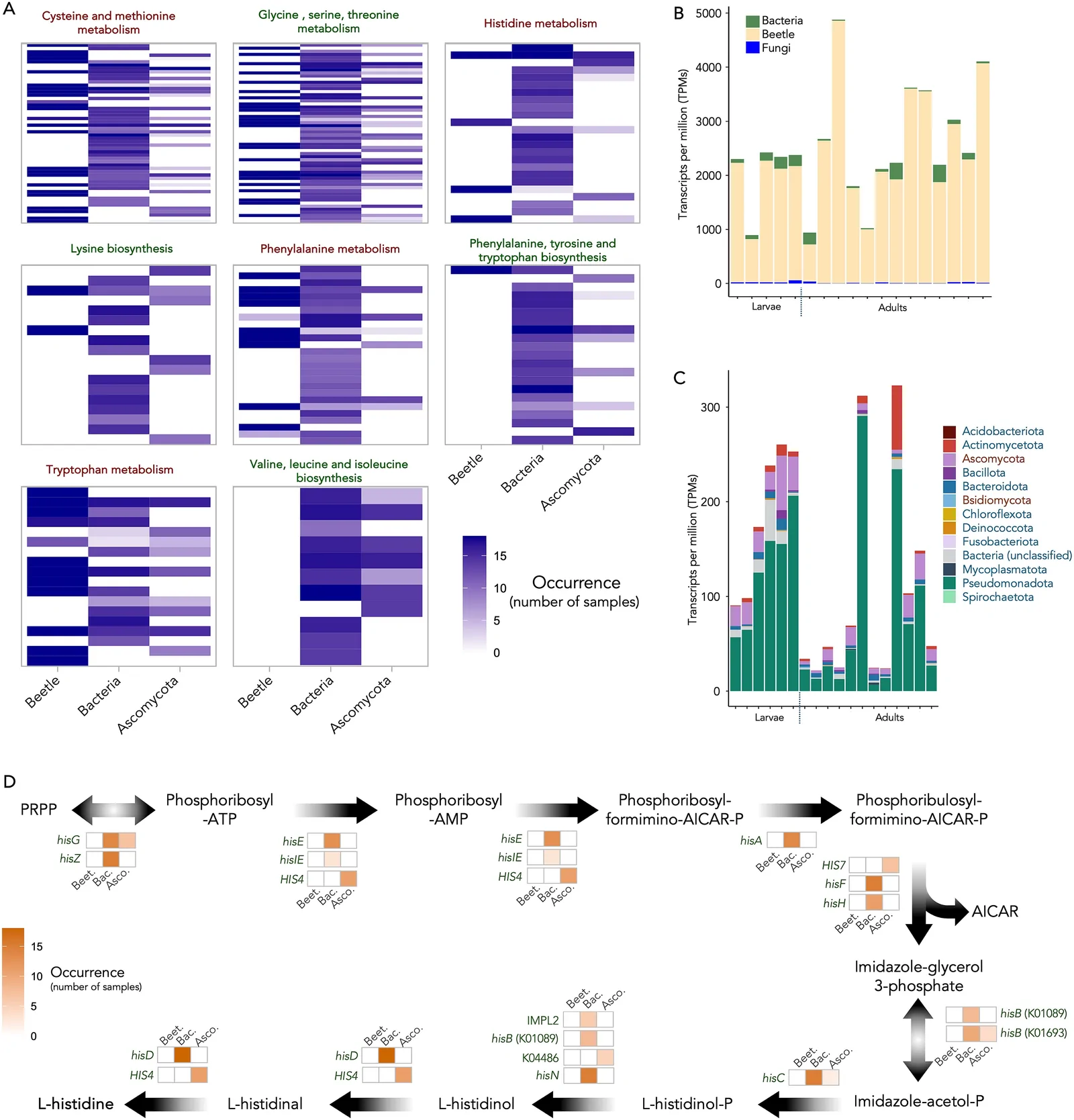
Interdependencies and co-metabolism in amino acid metabolism within the *Ips typographus* gut. (A) Contribution of different organisms (beetle, bacteria, or Ascomycota) to various steps in the amino acid metabolism. The heatmap shows the number of samples (*n* = 18) in which each function is active (occurrence). As shown, the beetle does not contribute to the biosynthesis of several amino acids. The specific details on the occurrence of each function can be observed in Supplementary Table 3. (B and C) Barplots showing the total contribution of each taxonomic group (B) or microbial phyla (C) to the overall amino acid metabolism. (D) Detailed example of how bacteria and Ascomycota contribute to histidine provision to the beetle holobiont. It can be shown that Ascomycota, which are not contributing to the fourth step on the route, likely rely on the bacterial metabolism to achieve a complete histidine metabolism.

Our metatranscriptomic data suggest a balanced metabolic collaboration in amino acid metabolism, with *I. typographus* actively contributing to the synthesis and interconversion of some amino acids, while microbial partners facilitate other pathways. Specifically, we focused on the biosynthesis and metabolism of essential amino acids such as cysteine, glycine, serine, threonine, histidine, lysine, phenylalanine, tyrosine, tryptophan, and branched-chain amino acids such as valine, leucine, and isoleucine (Fig. 4; Supplementary Table 4).

We identified several essential amino acids for which biosynthetic pathways were found exclusively in the symbiotic partners. For example, histidine biosynthesis appears to be largely completed by bacteria (Fig. 4; Supplementary Table 4). Although saccharomycetous fungi possess the genetic capacity for several key steps of this anabolic pathway (Supplementary Material), important differences were observed among taxa. The genome of *Cryptococcus porticicola* CCF 6641 encodes all enzymes required for histidine biosynthesis, whereas the genomes of *Kuraishia molischiana* CCF 6642, *Nakazawaea ambrosiae* CCF 6643, *Ogataea ramenticola* CCF 6644, and *Wickerhamomyces bisporus* CCF 6645 lack the sixth step, catalyzed by imidazoleglycerol-phosphate dehydratase (*hisB*; EC 4.2.1.19). In addition, transcription of the fourth step—the isomerization of phosphoribosyl-formimino-AICAR-P to phosphoribulosyl-formimino-AICAR-P—was not detected in the fungal transcript annotations (Fig. 4; Supplementary Table 4). This reaction is catalyzed by the HIS6 protein (*hisA* gene), which is present in the genomes of all analyzed yeasts (Supplementary Material) but appears not to be expressed under the conditions assessed, whereas it is actively expressed in bacteria and is essential for histidine biosynthesis [85]. Consistent with these observations, transcripts assigned to *hisB* were detected in only 4 of the 18 metatranscriptomic samples and were associated with Sordariomycetes (one sample) and Saccharomycetes (three samples). Together, these results suggest that the fungal contribution to histidine biosynthesis is taxon and condition dependent, whereas bacterial symbionts provide a more consistent capacity for maintaining histidine production within the bark beetle gut microbiota.

Lysine biosynthesis is supported by both bacterial (e.g. lysine 2-oxoglutarate transaminase (K00290)) and fungal contributions (Fig. 4; Supplementary Table 4). The pathways for the synthesis of branched-chain amino acids, specifically valine, leucine, and isoleucine, show no expressed genes in the bark beetle (Fig. 4; Supplementary Table 4), while microbial transcripts associated with these pathways are consistently detected across our samples. The biosynthesis of phenylalanine, tyrosine, and tryptophan also reveals a similar trend, with only one enzyme (K00014; *aroE*—shikimate dehydrogenase; EC:1.1.1.25) expressed in the beetle, contrasted with the extensive contributions from bacteria and Ascomycota (Fig. 4; Supplementary Table 4). On the contrary, the metabolism of glycine, serine, and threonine, as well as cysteine and methionine, are similarly characterized by strong contributions from the beetle and its symbionts.

The key microbial contributors in amino acid provision are *Pseudomonadota* (e.g. *Erwinia, Delftia, Phyllobacterium, Sphingomonas, Stenotrophomonas*, and *Pseudoxanthomonas*), *Actinomycetota* (e.g. *Cutibacterium* and *Rhodococcus*), and Ascomycota (e.g. Sordariomycetes, including *Ophiostoma*; Saccharomycetales, including *Ogataea*; and Pezizomycetes) (Fig. 4B, C).

### Signatures of vitamin co-metabolism in the bark beetle holobiont

**Figure 5:**
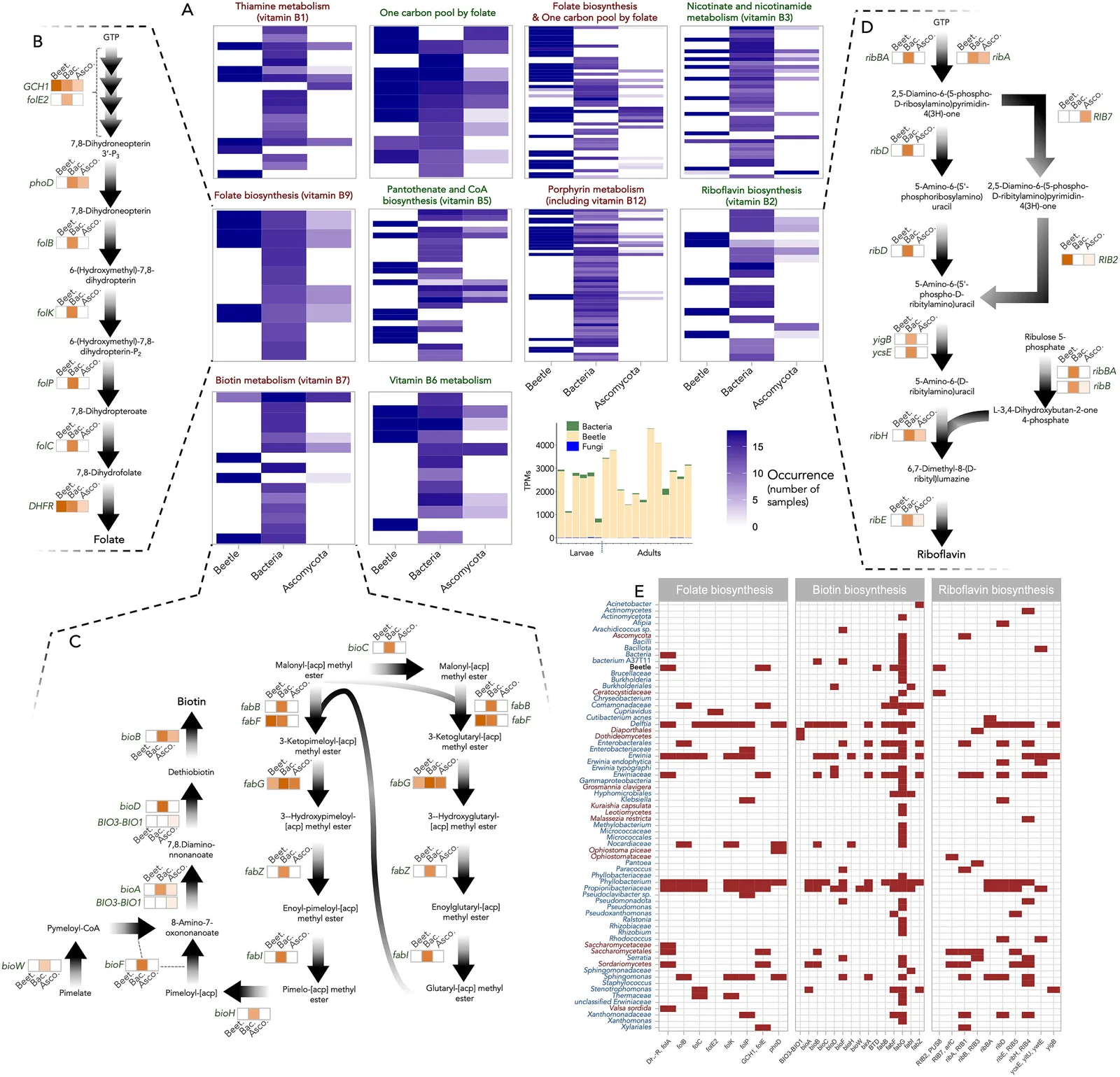
Interdependencies and co-metabolism in vitamins metabolism within the *Ips typographus* gut. (A) Contribution of different taxonomic groups (beetle, bacteria, or Ascomycota) to various steps in the amino acid metabolism. The heatmap shows the number of samples (*n* = 18) in which each function is active (occurrence). As shown, the beetle does not contribute to the biosynthesis of several vitamins. The specific details on the occurrence of each function can be observed in Supplementary Table 4. The barplots show the total contribution of each organism to the overall vitamin metabolism. (B–D) Detailed example of how bacteria and Ascomycota contribute to folate, biotin, and riboflavin provision to the bark beetle holobiont. These pathways show several cross-domain contributions (co-metabolism and interdependencies), with incomplete pathways in the eukaryotic counterparts that might be either contributing to the bacterial metabolism, which supply these vitamins to the holobiont. (E) Diagram showing the organisms (classified to the most precise annotated taxonomic rank) contributing to each function represented in panels (B)–(D).

The analysis of our metatranscriptomic data revealed the intricate division of labor between bacteria, Ascomycota, and the host beetle in the biosynthesis of essential vitamins such as folate (B_9_), biotin (B_7_), nicotinate, and nicotinamide (B_3_), pantothenate and coenzyme A (B_5_), riboflavin (B_2_), thiamine (B_1_), and vitamin B_6_ (Fig. 5A; Supplementary Table 5). For instance, we found that folate can be produced and utilized by the beetle and fungi, possibly by scavenging the vitamin B_9_ derivative 7,8-dihydrofolate produced by bacterial partners (Fig. 5B, Supplementary Table 5). Similarly, even though biotin is likely being used by the beetle and later recycled from biocytin through the biotinidase action (Supplementary Table 5), the biosynthesis of this vitamin appears to be driven by microbial symbionts (Fig. 5C). The bacterial biotin biosynthesis could also benefit from a few unspecific metabolic reactions that succeeded in the eukaryotic partners (Fig. 5C).

We also found that the biosynthesis of other vitamins is likely in charge of the bacterial symbionts and later used by the whole holobiont, such as vitamin B_6_, which is produced by bacteria, although the host and Ascomycota are modulating its chemical form among pyridoxal, pyridoxamine, and pyridoxine (Fig. 5A; Supplementary Table 5). Thiamine biosynthesis is active exclusively in bacteria (Fig. 5A; Supplementary Table 5). Finally, bacteria could benefit from a few host- or Ascomycota-produced intermediate metabolic steps in the biosynthesis of riboflavin (i.e. scavenging the RIB2 activity from the eukaryotic partners) (Fig. 5D; Supplementary Table 5). To corroborate the ability of *I. typographus* symbionts to aid in the vitamin provision, we analyzed the genomes of several *Erwinia* isolates and confirmed that they encode the complete riboflavin biosynthesis pathway (Supplementary Material). In addition, these isolates produced riboflavin *in vitro* (Supplementary Table 6). However, none of the genomes of the fungal isolates encode the complete riboflavin biosynthetic pathway from GTP (Supplementary Material).

Overall, the major vitamin-related biosynthetic activity is related to the beetle metabolism (Fig. 5A), although the reduced, but still important, microbial roles in these pathways are driven by *Pseudomonadota* (e.g. *Erwinia, Delftia, Phyllobacterium, Sphingomonas*), *Actinomycetota* (e.g. *Propionibacteriaceae*—including *Cutibacterium*—and *Rhodococcus*), and Ascomycota (e.g. Sordariomycetes, including *Ophiostoma*; Saccharomycetales, including *Ogataea*; and Pezizomycetes) (Fig. 5E). Nevertheless, many different microorganisms contribute to vitamin metabolism (Fig. 5E).

### Differential functional activity across life stages

Although our main focus is on functions that show cooperative expression across holobiont members, we also investigated whether other microbial metabolic processes exhibited life stage–specific activation patterns. To this end, we performed differential expression and KEGG enrichment analyses across larval, teneral adult, and parental adult stages, focusing exclusively on microbial genes to uncover additional biological processes potentially influenced by host development or changes in the symbiotic community.

**Figure 6:**
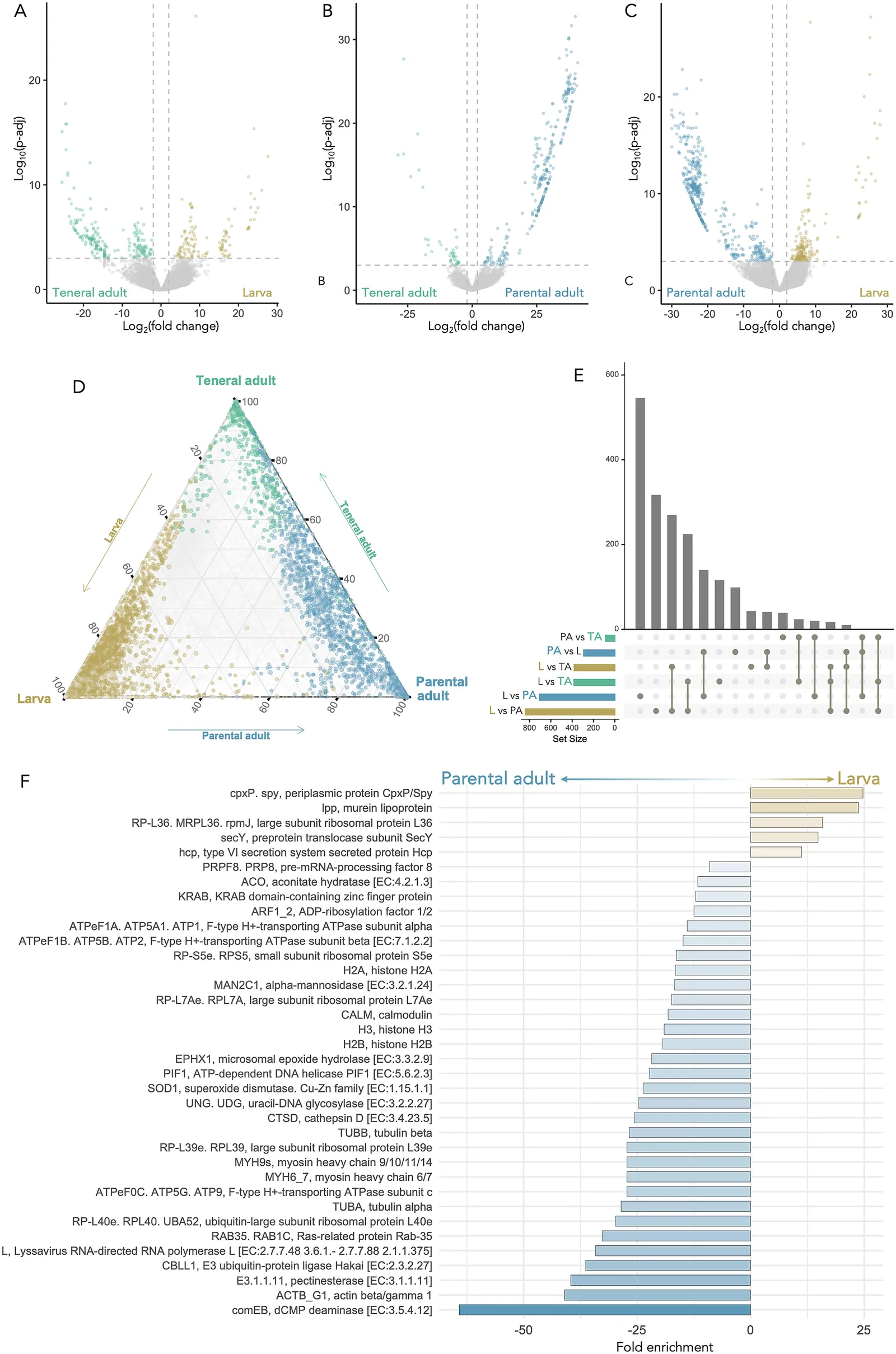
Differential expression of microbial genes in the gut of *Ips typographus* across developmental stages. (A–C) Volcano plots showing differentially expressed genes (DEGs) between larvae and teneral adults, teneral adults and parental adults, and parental adults and larvae. Genes more abundant in one condition are colored accordingly. (D) Ternary plot summarizing the relative expression of microbial genes across the three developmental stages. Each point represents a gene, positioned according to its normalized mean expression in larvae (L), teneral adults (TA), and parental adults (PA). Genes were assigned to a single differentially abundant group (DAA) if they showed a significant adjusted *P* value (<.05) in at least one pairwise contrast; the assignment reflects the first contrast meeting this threshold. Point color indicates the DAA group, size reflects the average expression level (log-transformed). Genes near a given vertex are dominated by expression changes specific to that comparison. Genes lying between two vertices show overlapping expression shifts, and those near the center reflect similar magnitudes across all comparisons. Genes not significantly differentially expressed in any comparison were colored in gray to distinguish them from DEGs. (E) UpSet plot showing intersections among DEGs (with padj <.05 and |log2FC| > 1) from each pairwise comparison. Bars are labeled with the condition where each gene is more abundant (in bold and color coded). L = larvae; TA = teneral adult; PA = parental adult. (F) Significantly enriched KEGG Orthology (KO) terms in the PA vs. L comparison—the most divergent developmental stages—are shown using their corresponding gene/protein annotations to facilitate biological interpretation.

Differential expression analysis revealed widespread transcriptional shifts between larvae and adults, particularly between larvae and parental adults (Fig. 6A–E). However, given the functional redundancy often observed in microbiomes—where distinct taxa can fulfill equivalent roles—we complemented gene-level comparisons with pathway enrichment analyses based on KEGG Orthologs (KOs), excluding beetle-derived sequences (Fig. 6D, Supplementary Table 7).

Notably, none of the core symbiotic functions described earlier—such as cooperation in amino acid and vitamin biosynthesis, cell wall hydrolysis, or nitrogen metabolism—showed evidence of differential enrichment across life stages. Instead, larval microbiomes exhibited increased representation of genes related to protein folding and export (e.g. *cpxP, secY, bamB*), type VI secretion (*hcp*), stress responses (*ibpA, cspA*), and cell division and envelope remodeling (*zapA, lpp, nlpI, zipA*). In contrast, microbiomes of parental adults showed enrichment in functions related to DNA replication, transcriptional regulation, and translation (e.g. RNA polymerase subunits, ribosomal proteins, elongation factors), as well as mobile genetic elements.

## Discussion

**Figure 7:**
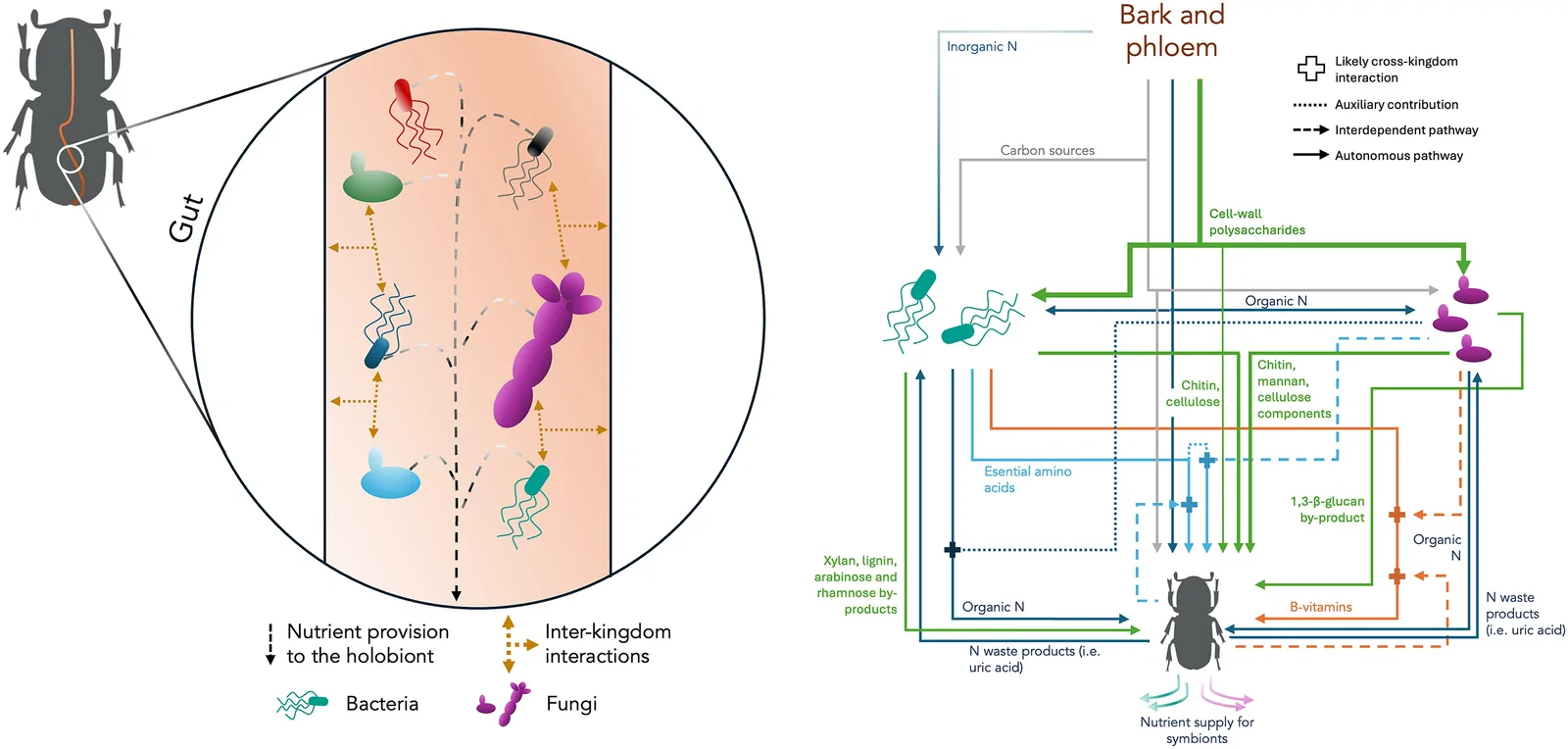
Schematic representation of cross-domain interactions within the bark beetle holobiont that drive nutrient provision for the host. The metabolic activities of the beetle, bacteria, and fungi are interconnected at several points, with events of co-metabolism and interdependencies likely facilitating the overall nutrient supply of the holobiont. On the right side of the figure, we highlight the main findings of our analyses, showing microbial contributions to the beetle holobiont, which often depend on or are supported by metabolic activities from other counterparts. The beetle also plays a role in supporting the nutrition of its microbiota. For simplicity, we focused on pathways contributing to *Ips typographus* nutrition, without detailing beetle-to-microbe contributions or fungal–bacterial cross-feeding beyond auxiliary aid events.

Our results highlight the critical role of microbial symbionts in the metabolic complementarity of the *I. typographus* holobiont, which may promote its fitness under the nutrient-poor conditions of its phloem-based diet. Overall, we found that microbial symbionts are likely to facilitate host nutrition (Fig. 7). While these findings suggest a role for microbial symbionts in host nutrition, it is also important to consider that some of these microbial activities, rather than acting solely for host benefit, may primarily serve the microbes themselves, supporting the synthesis of their own biomass, which can subsequently be utilized by the beetle through microbial turnover or digestion. Even though the bacterial partners in most of the examples presented were able to take over the entire pathway, we found evidence of cross-domain cooperation where eukaryotic functions seems to be interconnected or dependent on prokaryotic pathways (Fig. 7). Histidine biosynthesis provides a representative example. Reanalysis of previously reported genomes [39] revealed substantial variation among fungal symbionts: whereas *Cryptococcus porticicola* encodes a complete histidine biosynthetic pathway, several saccharomycetous yeasts lack key enzymes, including imidazoleglycerol-phosphate dehydratase (*HisB*). Furthermore, metatranscriptomic data showed that some pathway genes were not actively expressed despite being present in fungal genomes. In particular, transcripts corresponding to the fourth step of the pathway were not detected in fungal annotations, and expression of *hisB* was only sporadically observed across samples. In contrast, bacterial transcripts covering these missing or weakly expressed functions were consistently detected, suggesting that bacteria provide a more reliable capacity for histidine biosynthesis within the gut microbiota.

More broadly, we found that several ESBB fungal symbionts possess the genomic potential to synthesize amino acids and B vitamins, yet metatranscriptomic analyses indicate that not all of these pathways are transcriptionally active. This pattern suggests that fungi may down-regulate energetically costly biosynthetic functions and instead rely on nutrient acquisition from the environment or from other members of the microbial community when such resources are available. These findings emphasize the importance of distinguishing between the genomic potential and the actual metabolic activity in ecological contexts [41, 86], as metabolic interactions among symbionts may drive the observed phenotypes more than the genomic content alone. Although such examples of cooperative metabolism have previously been described in a few different niches [87, 88], the partial and incomplete contribution to a particular pathway is not widely described in ecological studies [89].

In support of our transcriptomic evidence, previous functional assays showed that >60% of ESBB bacterial isolates exhibited pectinase activity, nearly 57% could hydrolyze starch, and ~45% showed cellulolytic activity [30]. Although xylanase activity was less frequent, a subset of strains was able to degrade all four tested substrates (cellulose, xylan, starch, and pectin), highlighting the enzymatic versatility of some symbionts and reinforcing their potential contribution to nutrient processing within the holobiont. Consistent with our transcriptomic findings, previous functional assays demonstrated that some of the most relevant fungal taxa in *I. typographus*, such as a species of *Ogataea*—which also showed the highest expression of cell wall–degrading enzymes in our metatranscriptomic dataset—exhibit chitinase, β-mannosidase, β-xylosidase, cellobiohydrolase, and β-glucosidase activities [39]. These findings align with the detection of fungal expression of genes involved in starch degradation, and with genomic predictions indicating the presence of relevant pathways in *Ogataea ramenticola* CCF 6644 [39]. However, the same assays revealed that CCF 6644 did not degrade lignin or show detectable endo-1,4-β-d-glucanase activity, among other cell wall compounds’ catabolic activities, under *in vitro* conditions. This suggests that even enzymatically versatile fungi may lack the full capacity to degrade certain plant polymers on their own, reinforcing the idea that microbial cooperation is essential for the complete processing of complex substrates within the holobiont.

Our in-depth analysis of the degradation of plant and fungal cell wall polymers shows how different microbial partners may provide different enzymatic capabilities, enabling synergistic degradation of complex substrates. The observation that chitin degradation is a shared function across bacteria, fungi, and the beetle itself exemplifies the flexibility of the holobiont to access essential nutrients. Similarly, we found several biosynthetic pathways simultaneously activated in different bacterial taxa. Functional redundancy has been proposed as an adaptive mechanism to ensure nutrient availability under fluctuating environmental conditions [90–92]. However, despite the growing relevance of adaptive gene loss in recent research [93–96], it remains unclear how microbiomes orchestrate the “switching off” of functions that become redundant within the holobiont. Even though we found these redundant functions as active at the same niche, the pooling method of the sequenced samples, which was required to increase the input biomass, prevented any conclusion in that regard, reinforcing the need for spatial metatranscriptomics in host microbiomes to fully address such ecological theories [97]. Moreover, we acknowledge that adult beetles were not sexed prior to RNA extraction, and therefore, any potential sex-specific differences in microbial composition or transcriptional activity could not be addressed. Given that sex-based variation in insect microbiomes and nutritional requirements is well documented [98–100], this constitutes a limitation of the present study and may account for part of the variability observed in our data.

The exclusive role of Ascomycota in glucan degradation and the significant bacterial involvement in xylan and pectin degradation suggest a division of labor among symbionts, reducing functional overlap and promoting efficient resource utilization [101]. Similarly, bacteria are also potentially responsible for the biosynthesis of certain vitamins. Due to their importance for the holobiont, microbial taxa providing non-redundant metabolic activities could be crucial for understanding the phylosymbiotic features of the ESBB microbiome [29, 31, 102]. Such metabolic dependencies could indeed inspire biocontrol opportunities by targeting key symbiotic functions in the holobiont metabolic network [18, 103, 104].

Our analysis of glycoside hydrolase family 48 (GH48) enzymes suggest a role for the beetle in cellulose degradation. Even though GH48 has traditionally been associated with exoglucanase activity [81, 82], in our data these enzymes are annotated as endoglucanases [83, 84]. Also, end-acting glucanase action was reported in the ESBB genome, but just based on the GH48 annotation [80]. This unexpected endoglucanase capacity raises important questions about the evolution of functional plasticity within GH families and the beetle’s potential to independently contribute to cellulose catabolism. Experimental validation of this enzymatic activity could provide further insights into the beetle’s metabolic independence and its role within the holobiont.

Nitrogen is a limiting element, and within the context of the bark beetle holobiont, discussions have primarily focused on organic nitrogen derived from the phloem, utilized by both the beetle and associated microbes [20]. Our data reveal the expression of complete pathways for the acquisition of inorganic nitrogen in form of nitrogen oxides by bacteria. Conversely, we did not observe the capability to fix molecular nitrogen (N_2_). Nitrates and nitrites accumulate in plant cells, including the bark of conifers [105], entering from surrounding water and atmospheric deposition. This discovery introduces a previously unconsidered pathway for nitrogen entry into the holobiont, necessitating further verification. It also suggests that an increased supply of inorganic nitrogen, potentially due to forest collapse and air pollution [106], could enhance nitrogen availability for the beetle, thereby potentially increasing its fitness.

We found that bacteria such as *Erwinia, Delftia, Phyllobacterium, Sphingomonas, Serratia, Cupriavidus, Stenotrophomonas, Pseudoxanthomonas, Cutibacterium, Rhodococcus*, and *Pantoea* and fungi such as *Ophiostoma, Ogataea, Cyberlindnera*, and *Kuraishia* play key roles in the nutrient cycling of the beetle holobiont. Most of these taxa have been identified as core microbial genera in the ESBB gut microbiome using DNA-based methods [29–35]. Specially, *Erwinia* spp. have been frequently associated with both bark and ambrosia beetles [29, 30, 107, 108]. However, some of these taxa have not received much attention in terms of bark beetle biology. The most notorious example is *Delftia*, a genus actively involved in the nitrogen cycle, amino acid and vitamin supply, and polysaccharide catabolism, but which has not been detected as an abundant taxon in the ESBB communities [29–35]. Therefore, we encourage attempts to isolate and characterize *Delftia* strains from *I. typographus* to provide more resources for understanding their ecological characteristics. In contrast, the genus *Pseudomonas* has been proposed as a ubiquitous taxon not only in the ESBB microbiome [29, 30, 109] but also in several bark beetle species [23]. However, we found no evidence for their involvement in the nutrient cycles analyzed in this work.

Although our study centered on nutritional functions due to their clearer annotation and ecological relevance, other microbial roles such as detoxification of plant defense compounds and potential virulence toward the host plant remain unexplored. Future work should aim to overcome current annotation limitations to investigate these important functional traits and better understand the full spectrum of holobiont interactions.

The high diversity of viral transcripts observed in the dataset presents an intriguing, yet unresolved, aspect of the *I. typographus* holobiont. Although their functional roles remain speculative, viral elements could influence microbial community dynamics [110–112]. Further investigation into the functional contributions of these viral communities may reveal their role in shaping the metabolic and ecological adaptability of the holobiont. Indeed, we also provide evidence of a likely nutrient exchange among the host and the symbionts, which is a frequent event in natural microbial communities [113]. However, the specifics of these exchanges, including spatial proximity and the mechanisms of transfer, remain unresolved in our data. Recent research has demonstrated that vitamin exchange among marine bacteria can require cell lysis prior to uptake [114]. Given that some of the suggested metabolic interdependencies in the ESBB microbiome involve intermediary metabolites from larger pathways, we hypothesize that such nutrient exchanges could also be facilitated by cell lysis, potentially mediated by viruses. We also found mycoviruses in the beetle gut. Some mycoviruses are known to shape fungal ecology as they moderate fungal pathogenicity [115–117]. Recently, mycoviruses were identified in *Leucoagaricus gongylophorus*, a fungus farmed by leafcutter ants, having potential impact on development of fungal-ant symbiosis [112]. The role of mycoviruses in the development of *I. typographus* waits to be explored in further research. On the other hand, small, readily available metabolites such as amino acids or nitrogen-containing molecules, which can be transported across cell membranes, are commonly shared between species or subpopulations as part of cross-feeding strategies [118–120]. Also, extracellular vesicles might play a key role in these interactions by enabling more targeted transfer of molecules [121, 122]. Notably, while many of cross-feeding interactions appear cooperative, these relationships can also be vulnerable to exploitation, where certain taxa benefit from shared metabolites without contributing to their production [120]. Beyond these microbe–microbe exchanges, it is also possible that the host exerts some level of control over these interactions. For instance, *I. typographus* might influence the structure or activity of its microbiome by modulating gut conditions, regulating metabolite access, or promoting certain microbial partners through immune tolerance or spatial organization. Although speculative, such regulatory roles could contribute to the stabilization of key metabolic interactions within the holobiont. Similar patterns of metabolic complementarity and nutrient exchange have also been reported in other holobiont systems, including deep-sea tubeworms, termites, and leafcutter ants [123–125], suggesting that such interactions may represent a widespread strategy for overcoming nutritional limitations.

The differential expression patterns observed between larval and adult stages reveal dynamic shifts in microbial metabolic activity throughout the beetle’s development. In larvae, the higher representation of genes related to growth, stress response, and microbial interaction suggests a microbial community more engaged in active growth, structural remodeling, and microbial competition or host interaction. In contrast, the transcriptomic profile of the gut microbiome found in parental adults is enriched for functions related to maintenance, transcriptional regulation, and horizontal gene transfer. This pattern may reflect lower cell division rates but increased maintenance, signaling, or gene mobility within the microbial community at this stage. These findings indicate that, beyond essential cooperative functions, the microbiota exhibits functional plasticity that may align with the host’s developmental requirements.

Overall, our findings provide strong evidence of metabolic complementarity and functional integration within the *I. typographus* holobiont, emphasizing the potential collaborative roles of bacteria, fungi, and the beetle in overcoming dietary limitations and providing clues to understand mutualistic symbioses. Our results support the idea that resource partitioning and metabolic cooperation are crucial strategies in the adaptation of holobionts to challenging ecological niches. This study not only advances our understanding of microbial cooperation in bark beetle ecosystems, but also lays the foundation for future research on the evolutionary drivers of metabolic specialization and the potential biotechnological applications of these metabolic pathways in lignocellulose degradation.

## Supplementary Material

Supplementary_material_wrag227

## Data Availability

All raw sequences obtained through metatranscriptomic sequencing are available at the NCBI Sequence Read Archive (SRA) database under the Bioproject no. PRJNA1216986. Genomic assemblies for bacterial isolates are available under the Bioproject no. PRJNA1474338.
