# Supplementary material for: Cross-domain cooperation drives nutrient acquisition and metabolism in the bark beetle holobiont
Source: ISME J. 2026 Sep 16;20(1):wrag227. doi: 10.1093/ismejo/wrag227 (PMC13615527; doi:10.1093/ismejo/wrag227)
Supplement: Supplementary file 1 [file supplementary_material_wrag227.zip › Supplemental information for review.docx]

**Supplemental information for review.**

**Supplementary Table 1**. Functions involved in cell wall polysaccharide degradation. The values indicate the total count of samples in which each function is active, categorized by taxonomic group (beetle, Bacteria and Ascomycota).

**Supplementary Table 2**. Functions involved in nitrogen metabolism. The values indicate the total count of samples in which each function is active, categorized by taxonomic group (beetle, Bacteria and Ascomycota).

**Supplementary Table 3**. Results for the *in vitro* tests used to prove the ability of microbial isolates to metabolize different nitrogen sources.

**Supplementary Table 4**. Functions involved in amino acid metabolism. The values indicate the total count of samples in which each function is active, categorized by taxonomic group (beetle, Bacteria and Ascomycota).

**Supplementary Table 5**. Functions involved in vitamin metabolism. The values indicate the total count of samples in which each function is active, categorized by taxonomic group (beetle, Bacteria and Ascomycota).

**Supplementary Table 6**. Results for the in vitro tests used to prove the ability of bacterial isolates to produce extracellular riboflavin.

**Supplementary table 7.** KEGG Orthology (KO) terms significantly enriched in the comparison between parental adults and larvae. Each row corresponds to a KO identifier with the associated enrichment statistics and annotations. “Group with higher expression” indicates the developmental stage in which genes assigned to the KO term are more highly expressed.
