# Supplementary material for: Cross-domain cooperation drives nutrient acquisition and metabolism in the bark beetle holobiont
Source: ISME J. 2026 Sep 16;20(1):wrag227. doi: 10.1093/ismejo/wrag227 (PMC13615527; doi:10.1093/ismejo/wrag227)
Supplement: Supplementary file 1 [file supplementary_material_wrag227.zip › Supplementary Material-R3.docx]

**Supplementary Material**

Cross-domain cooperation drives nutrient acquisition and metabolism in the bark beetle holobiont

**Zaki Saati-Santamaría^1,2,3,4*^, Tereza Veselská^1^, Karel Švec^1,5^, Martin Kostovčík^1^, Ezequiel Peral-Aranega^2,3^, Barbora Křížková^1^, Paula García-Fraile^2,3,4,6^, Miroslav Kolařík^1*^**

^1^ Laboratory of Fungal Genetics and Metabolism, Institute of Microbiology, The Czech Academy of Sciences, Prague, Czech Republic

^2^ Institute for Agribiotechnology Research (CIALE), Universidad de Salamanca, Salamanca, Spain

^3^ Departamento de Microbiología y Genética, Universidad de Salamanca, Salamanca, Spain.

^4^ Unidad de Excelencia Producción, Agrícola y Medioambiente (AGRIENVIRONMENT), Universidad de Salamanca, Salamanca, Spain.

^5^ The Department of Genetics and Microbiology, Charles University in Prague.

^6^Associated Research Unit of Plant-Microorganism Interaction, Universidad de Salamanca – IRNASA-CSIC, Salamanca, Spain.

***Corresponding authors:**

Dr. Zaki Saati-Santamaría ([zakisaati@usal.es](mailto:zakisaati@usal.es)), Microbiology and Genetics Department, University of Salamanca, Doctores de la Reina SN, Edificio Departamental de Biología, Salamanca, 37004, Spain.

Dr Miroslav Kolařík ([mkolarik@biomed.cas.cz](mailto:mkolarik@biomed.cas.cz)), Laboratory of Fungal Genetics and Metabolism, Institute of Microbiology of the Czech Academy of Sciences, Videnska 1083, Prague, 142 00, Czech Republic.

**Short title:** Cross-domain cooperation in bark beetles

**Keywords:** Holobiont; Host-microbe interactions; Co-metabolism; Symbiosis; Metatranscriptome; Cross-domain RNA-Seq; Bark beetle

This document contains supplementary results associated with the main manuscript. Here we present screenshots of metabolic pathway reconstructions derived from the genomes of the following strains: *Erwinia billingiae* AC7, *Erwinia typographi* CA1A and AC8, ‘*Erwinia* sp009765445’ MMI19, *Cryptococcus* *porticicola* CCF 6641, *Kuraishia molischiana* CCF 6642, *Nakazawaea ambrosiae* CCF 6643, *Ogataea ramenticola* CCF 6644, and *Wickerhamomyces bisporus* CCF 6645. The figures include pathways related to urate degradation (pages 2–3), as well as the biosynthetic pathways for riboflavin (pages 3–5) and histidine (pages 5-6). In all figures, green boxes represent enzymes encoded in the genomes, whereas white boxes indicate enzymes that are absent or not detected in the corresponding genomic annotations.

**Purine** metabolism, including **urate** (red dot) catabolism for bacterial strains *Erwinia billingiae* AC7, *Erwinia typographi* CA1A, and ‘*Erwinia* sp009765445’ MMI19.

**Purine** metabolism, including **urate** (red dot) catabolism for bacterial strain *Erwinia typographi* AC8.

**Purine** metabolism, including **urate** (red dot) catabolism for fungal strains *Cryptococcus* *porticicola* CCF 6641, *Kuraishia molischiana* CCF 6642, *Nakazawaea ambrosiae* CCF 6643, *Ogataea ramenticola* CCF 6644, and *Wickerhamomyces bisporus* CCF 6645.

**Riboflavin** biosynthetic pathway for bacterial strains *Erwinia billingiae* AC7, *Erwinia typographi* CA1A and AC8, and ‘*Erwinia* sp009765445’ MMI19.

**Incomplete Riboflavin** biosynthetic pathway for fungal strains:

*Cryptococcus* *porticicola* CCF 6641:

*Kuraishia molischiana* CCF 6642:

*Nakazawaea ambrosiae* CCF 6643:

*Ogataea ramenticola* CCF 6644:

and *Wickerhamomyces bisporus* CCF 6645:

**Histidine** biosynthetic pathway for fungal strain *Cryptococcus* *porticicola* CCF 6641:

**Incomplete Histidine** biosynthetic pathway for fungal strains:

*Kuraishia molischiana* CCF 6642 and *Nakazawaea ambrosiae* CCF 6643:

*Ogataea ramenticola* CCF 6644, and *Wickerhamomyces bisporus* CCF 6645:
